# Gestational age at birth and behavioral problems from four to 11 years of age: birth cohort study

**DOI:** 10.1186/s12887-017-0936-3

**Published:** 2017-08-23

**Authors:** Iná S. Santos, Fernando C. Barros, Tiago Munhoz, Alicia Matijasevich

**Affiliations:** 1 Program in Epidemiology, Federal University of Pelotas, Rua Marechal Deodoro 1160, 3o piso, Pelotas, RS 96020220 Brazil; 2 Program in Epidemiology, Federal University of Pelotas and Postgraduate Program in Health and Behaviour, Catholic University of Pelotas, Pelotas, RS Brazil; 3 Faculty of Psychology, Federal University of Pelotas, Pelotas, RS Brazil; 4 Department of Preventive Medicine, University of São Paulo, São Paulo, SP Brazil

**Keywords:** Behavioral problems, Psychiatric disorders, Internalizing disorders, Externalizing disorders, Childhood, Adolescence, Cohort study

## Abstract

**Background:**

Studies conducted mainly in high-income countries have shown that preterm births are associated with increased risk of behavioral problems and psychiatric disorders. The aim of this study was to assess the prevalence of behavioral problems from middle-childhood to early-adolescence according to gestational age at birth in a middle-income setting.

**Methods:**

A population-based birth cohort (*n* = 4231) in Pelotas, Brazil, was followed-up in several occasions from birth to 11 years. Estimated GA was based on last menstrual period or, when unknown or inconsistent, on the Dubowitz method. Behavioral problems were assessed at 4 (Child Behavior Checklist - CBCL), and at 6 and 11 years (Development and Well-Being Assessment - DAWBA) tool. Maternal socio-economic characteristics and depression at 2, 4 and 6 years post-partum, child perinatal characteristics and breastfeeding duration were used as confounders. Analyses were run by linear and logistic regression.

**Results:**

Three thousand two hundred four children had full information on gestational age, CBCL and DAWBA. At 4 years, mean total (42.9 ± 24.0) and mean externalizing (18.8 ± 9.1) CBCL scores were higher among preterm girls born at <34 weeks than among full term girls (33.2 ± 15.1 and 15.0 ± 6.6, respectively). After controlling for confounders the association was no longer significant. At the age of 6 years there was no association between gestational age and behavior, neither in crude nor in adjusted analyses. Odds ratio for any psychiatric disorders at 11 years was 60% (1.6; 1.1–2.1) higher among those born at 34–36 weeks than in full-term children, but the association disappeared in adjusted analyses.

**Conclusion:**

At this large cohort, behavioral problems from middle-childhood to early-adolescence are more related to family socio-economic characteristics and to other child perinatal conditions than to gestational age at birth.

## Background

The effect of gestational age at birth, particularly the effect of prematurity (gestational age < 37 weeks) and early prematurity (< 34 weeks) has been subject of several studies worldwide. [1–3] Although the majority of preterm babies survive without impairment, studies have shown that preterm births are associated with increased risk of neonatal and infant mortality, [4–6] besides of predicting offspring morbidity across lifespan, including psychiatric disorders, academic problems, and social difficulties [7, 8].

Worldwide, the preterm birth rates range from 5% in high-income countries to 25% in low- and middle-income countries. [9] Since 1990, prematurity rates are increasing in all countries with reliable time trend data [10]. According to the World Health Organization estimates preterm rates are highest in general for low-income countries (11.8%), followed by lower middle-income countries (11.3%) and lowest for upper middle- and high-income countries (9.4 and 9.3%, respectively) [7]. Despite the fact that most of the preterm births take place in low- and middle-income countries, the available literature on the consequences of gestational age over behavioral problems later in life comes mainly from studies carried out in high-income countries and the results of the studies are inconsistent [1].

In Brazil, one of the top 10 countries with the highest numbers of preterm births worldwide, [7] the prevalence of psychiatric disorders in childhood, adolescence and adulthood is higher than in high-income countries, [11–15] turning it a suitable place to explore the association between prematurity and mental health. This study was planned to compare the prevalence of internalizing and externalizing symptoms at four, six and 11 years of age according to gestational age at birth among children from a population-based birth cohort.

## Methods

From January 1st to December 31st, 2004, all live births of mothers delivering at the maternity hospitals in Pelotas, a Southern Brazilian city, and residing at the urban area of the city were eligible to the Pelotas 2004 Birth Cohort. More than 99% of all deliveries took place in one of the five maternity hospitals available in the city. Mothers were interviewed soon after delivery regarding demographic, socio-economic, behavioral and biological characteristics, reproductive history, and health care services utilization. The non-response rate at recruitment was below 1%. A total of 4231 live births were enrolled in the cohort. Follow-ups were done at home at mean ages 3.0 ± 0.1, 11.9 ± 0.2, 23.9 ± 0.4 and 49.5 ± 1.7 months, and at a research clinic at 6.8 ± 0.3 and 10.9 ± 0.3 years, with follow-up rates between 86 and 96%. A detailed description of the methodology is given elsewhere [16].

Estimates of gestational age were based on the last menstrual period (LMP) providing they were consistent with predicted birth weight, length, and head circumference, based on the normal curves for these parameters for each week of gestational age. [17] If LMP-based gestational age was unknown or inconsistent, the clinical maturity estimate based on the Dubowitz method, [18] which was performed on almost all newborns, was adopted. Gestational age was categorized as extremely preterm (< 28 weeks), very preterm (28–31 weeks), moderate preterm (32–33 weeks), late preterm (34–36 weeks of gestations), and full term newborns (≥ 37 weeks) [19].

Adverse behavioral outcomes were assessed at four, six and 11 years of age. At the four-year follow-up children were assessed for the presence of behavioral/emotional problems by the application of the Child Behavioral Checklist (CBCL) to mothers or caregivers. [20] The instrument provides scores on eight scales: withdrawn, somatic complaints, anxious/depressed, social problems, thought problems, attention problems, aggressive behavior and rule-breaking behavior. Data from these scales were summed to provide an overall score (total problems) and two broad dimensions (internalizing and externalizing problems). The CBCL was validated among Brazilian children [21].

At the six and 11-year follow-ups children were assessed using the Development and Well-Being Assessment (DAWBA) tool, [22] an instrument designed to assess psychiatric disorders according to The International Classification of Diseases-Tenth Revision (ICD-10), The Diagnostic and Statistical Manual of Mental Disorders-4th Edition, and The Diagnostic and Statistical Manual of Mental Disorders-Fifth Edition criteria for ages 5–17 years. [23, 24] The instrument was validated in the Brazilian population. [25] Trained psychologists administered the DAWBA to mothers or caregivers. A computerized algorithm provided the probability of a child having any psychiatric disorder based on responses to the structured questions, but clinical Raters (two experienced child psychiatrists) provided the final evaluation. The Raters reviewed the symptoms and the negative impact of symptoms over the child relationship within the family and with persons from the social environment, as well as all the qualitative information in their own assessment [26]. Externalizing disorders included oppositional defiant disorder, conduct disorder and any Attention Deficit Hyperactivity Disorder **(**ADHD), including hyperactive, inattentive and combined sub-types and ADHD not otherwise specified. Internalizing disorders included diagnoses of anxiety and depression. For comparability, in the present analyses the ICD-10 criteria were used.

Information on family income in the month prior to delivery; maternal schooling, age, skin color, living with a partner, parity, smoking during pregnancy, depression (assessed at 2-, 4- and 6-year follow-ups and defined as probable when the Edinburgh Post-natal Depression Score was ≥13), [27] and type of delivery were collected. At birth, child sex, low birth weight (birth weight < 2500 g), Apgar score at 1st and 5th minutes, and need of intensive care were recorded. Information on hospital admissions in the first year and breastfeeding duration (<12, 12–23.9 and ≥24 months) was gathered.

Only single births were included in the analyses. Due to the small number of children born at <34 weeks, the extremely, very and moderate preterm births were grouped into <34 weeks for the analyses. The CBCL scores were analyzed as continuous outcome in original score units (a higher score reflects greater problems). To assess the association between gestational age and CBCL scores ANOVA analysis was conducted. Multivariate linear regression was used to estimate the effect of gestational age at birth over total, internalizing and externalizing problems at age 4 years adjusting for potential confounding factors in separate models. Potential confounders were grouped and included in the adjusted analysis using a backward strategy selection. If the significance level was below 0.20 the variable remained in the model as a potential confounder for the next level [28].

Psychiatric disorders at six and 11 years (any psychiatric disorder, internalizing and externalizing disorders) were analyzed as dichotomous outcomes. Pearson *X* ^2^ tests were used to assess the association between gestational age and the outcomes. Crude and adjusted analyses were carried out with logistic regression. Because there was no effect modification by child sex, the adjusted linear and logistic regression analyses were carried out with the whole sample, having child sex as a confounding variable.

## Results

A total of 3204 children were followed-up from birth to 11 years of age and had full information on gestational age at birth, CBCL at four years and DAWBA at six and 11 years of age. Among them, 416 (12.9%) were preterm. Prevalence of late preterm (34–36 weeks) and preterms <34 weeks was 10.6 and 2.3%, respectively.

Tables 1 and 2 show that gestational age groups were different in regard to several background characteristics. Preterm births were more prevalent among low-income families and among non-white, adolescent mothers with lower level of formal education (Table 1). In Table 2, prevalence of low Apgar at 1st and 5th minutes and low birth weight were higher among preterm newborns that also required intensive care more frequently than the full-term newborns. Breastfeeding was interrupted earlier and hospitalizations during infancy were more frequent among preterm than among term infants. There was no difference in prevalence of maternal depression at 24 months and at 6 years after delivery between the different gestational ages, whereas depression was higher among mothers of preterm born children at the 48-month follow-up.

**Table 1:** Distribution of gestational age at birth according to maternal characteristics among singleton pregnancies, Pelotas, Brazil (*n* = 3204)

Maternal characteristics	All n	<34 n (%)	34–36 n (%)	37+ n (%)	p-value^b^
Family income (MW)^a^					0.002
< =1	615	20 (3.3)	94 (15.3)	501 (81.5)	
1.1–3	1524	37 (2.4)	146 (9.6)	1341 (88.0)	
3.1–6	743	11 (1.5)	74 (10.0)	658 (88.6)	
6.1–10	171	2 (1.2)	15 (8.8)	154 (90.1)	
> 10	145	5 (3.5)	12 (8.3)	128 (88.3)	
Maternal schooling (years)					0.002
0	26	0 (0)	1 (3.9)	25 (96.2)	
1–4	440	16 (3.6)	63 (14.3)	361 (82.1)	
5–8	1324	36 (2.7)	150 (11.3)	1138 (86.0)	
≥ 9	1382	22 (1.6)	125 (9.0)	1235 (89.4)	
Maternal skin color					0.007
White	2346	51 (2.2)	233 (9.9)	2062 (87.9)	
Black	642	22 (3.4)	88 (13.7)	532 (82.9)	
Other	216	2 (0.9)	20 (9.3)	194 (89.8)	
Single mother					0.851
Yes	497	13 (2.6)	55 (11.1)	429 (86.3)	
No	2707	62 (2.3)	286 (10.6)	2359 (87.1)	
Maternal age (years)					0.005
< 20	604	21 (3.5)	83 (13.7)	500 (82.8)	
20–34	2140	41 (1.9)	219 (10.2)	1880 (87.9)	
> 34	459	13 (2.8)	39 (8.5)	407 (88.7)	
Parity					0.144
0	1266	34 (2.7)	153 (12.1)	1079 (85.2)	
1	859	15 (1.8)	85 (9.9)	759 (88.4)	
≥ 2	1078	26 (2.4)	103 (9.6)	949 (88.0)	
Maternal smoking during pregnancy					0.128
No	2344	49 (2.1)	240 (10.2)	2055 (87.7)	
Yes	860	26 (3.0)	101 (11.7)	733 (85.2)	
Type of delivery					0.827
Vaginal	1760	41 (2.3)	182 (10.3)	1537 (87.3)	
Cesarean section	1444	34 (2.4)	159 (11.0)	1251 (86.6)	

^a^MW: minimum wages

^b^Pearson *X* ^2^ test

**Table 2:** Child’s characteristics in the first year of life and maternal depression at 24 and 48 months and 6 years after the child birth, Pelotas, Brazil (*n* = 3204)

Variable	All	<34	34–36	37+	*p*-value^b^
Sex					0.605
Male	1654	43 (2.6)	176 (10.6)	1435 (86.8)	
Female	1550	32 (2.1)	165 (10.7)	1353 (87.3)	
Apgar 1st minute <7					<0.001
No	2813	49 (1.7)	278 (9.9)	2486 (88.4)	
Yes	371	26 (7.0)	60 (16.2)	285 (76.8)	
Apgar 5th minute <7					<0.001
No	3138	66 (2.1)	328 (10.5)	2744 (87.4)	
Yes	49	9 (18.4)	11 (22.5)	29 (59.2)	
Hospitalization in intensive care unit at birth					<0.001
No	2925	25 (0.9)	256 (8.8)	2644 (90.4)	
Yes	271	50 (18.5)	83 (30.6)	138 (50.9)	
Low birth weight					<0.001
No	2949	11 (0.4)	245 (8.3)	2693 (91.3)	
Yes	255	64 (25.1)	96 (37.7)	95 (37.3)	
Breastfeeding duration (months)					0.009
< 12	1954	61 (3.1)	205 (10.5)	1688 (86.4)	
12–23.9	531	7 (1.3)	60 (11.3)	464 (87.4)	
≥ 24	716	7 (1.0)	76 (10.6)	633 (88.4)	
Hospitalization in the first year of life					<0.001
No	2578	26 (1.0)	237 (9.2)	2315 (89.8)	
Yes	557	47 (8.4)	95 (17.1)	415 (74.5)	
Maternal depression (EPDS ≥13) (24 months after birth)^a^					0.061
No	2651	66 (2.5)	268 (10.1)	2317 (87.4)	
Yes	488	9 (1.8)	66 (13.5)	413 (84.6)	
Maternal depression (EPDS ≥13) (48 months after birth)^a^					0.002
No	2649	65 (2.5)	259 (9.8)	2325 (87.8)	
Yes	546	10 (1.8)	81 (14.8)	455 (83.3)	
Maternal depression (EPDS ≥13)					0.193
(6 years after birth)					
No	2406	58 (2.4)	245 (10.2)	2103 (87.4)	
Yes	469	11 (2.4)	61 (13.0)	397 (84.7)	

^a^ *EPDS* Edinburgh Post-natal Depression Scale

^b^Pearson *X* ^2^ test

In Table 3, mean CBCL total score was higher among girls born at <34 weeks (42.9 ± 24.0) and at 34–36 weeks (35.7 ± 17.9) than among girls born full term (33.5 ± 15.9) (*p* = 0.001). Mean externalizing scores were also higher among preterm than among full-term girls. Among boys, mean internalizing problems score was higher among those born at 34–36 weeks (6.8 ± 4.7) in comparison to those born at term (6.1 ± 4.3) (*p* = 0.045).

**Table 3:** Mean (Standard Deviation) CBCL scores at 48 months of age (total score, internalizing and externalizing problems) according to categories of gestational age among singleton pregnancies, Pelotas, Brazil

Gestational age (completed weeks)	Mean (SD) CBCL total score	*p*-value^a^	Mean (SD) Internalizing problems	*p*-value^a^	Mean (SD) Externalizing problems	*p*-value^a^
Girls						
		0.001		0.163		0.004
<34	42.9 (24.0)		7.5 (6.8)		18.8 (9.1)	
34–36	35.7 (17.9)		6.8 (5.3)		15.7 (7.5)	
37+	33.5 (15.4)		6.3 (4.6)		15.0 (6.6)	
All	33.9 (15.9)		6.4 (4.7)		15.1 (6.8)	
Boys						
		0.213		0.045		0.611
<34	35.3 (10.3)		5.2 (2.7)		16.1 (5.9)	
34–36	37.1 (17.4)		6.8 (4.7)		16.1 (7.6)	
37+	34.8 (16.3)		6.1 (4.3)		15.6 (7.4)	
All	35.0 (16.3)		6.2 (4.3)		15.6 (7.4)	

^a^ANOVA test

In Table 4, after allowing for family income, maternal marital status, skin color, schooling, age, parity, and smoking during pregnancy, type of delivery, child sex, low birth weight, Apgar at 1st and 5th minutes, and intensive care hospitalization at birth, the association between gestational age and total CBCL score at age 4 years disappeared. In the same way, the association between preterm birth and internalizing and externalizing problems at 4 years observed in crude analyses, disappeared after adjusting to family income, and maternal marital status, skin color and schooling.

**Table 4:** Crude and adjusted coefficients for CBCL scores (total score, internalizing and externalizing problems) with standard errors (SE) at 4 years of age among singleton pregnancies (37+ weeks of gestational age = reference category)

Gestational age (completed weeks)	Models	Total CBCL		Internalizing problems		Externalizing problems	
		Beta (SE)	*p*-value	Beta (SE)	*p*-value	Beta (SE)	*p*-value
	Model 1^a^		0.003		0.002		0.002
<34		4.4 (1.9)		−0.03 (0.5)		2.0 (0.8)	
34–36		2.2 (0.9)		0.6 (0.3)		0.6 (0.4)	
37+		0.0 (reference)		0.0 (reference)		0.0 (reference)	
	Model 2^b^		0.027		0.170		0.076
<34		3.7 (1.8)		−0.2 (0.5)		1.7 (0.8)	
34–36		1.7 (0.9)		0.5 (0.3)		0.4 (0.4)	
37+		0.0 (reference)		0.0 (reference)		0.0 (reference)	
	Model 3^c^		0.051		0.204		0.117
<34		3.3 (1.8)		−0.3 (0.5)		1.6 (0.8)	
34–36		1.5 (0.9)		0.4 (0.3)		0.3 (0.4)	
37+		0.0 (reference)		0.0 (reference)		0.0 (reference)	
	Model 4^d^		0.336		0.188		0.472
<34		2.0 (2.1)		−0.5 (0.6)		1.1 (0.9)	
34–36		1.3 (0.9)		0.4 (0.3)		0.2 (0.4)	
37+		0.0 (reference)		0.0 (reference)		0.0 (reference)	
	Model 5^e^		0.356		0.263		0.382
<34		2.5 (2.1)		−0.5 (0.6)		1.3 (0.9)	
34–36		1.0 (1.0)		0.3 (0.3)		0.1 (0.4)	
37+		0.0 (reference)		0.0 (reference)		0.0 (reference)	

^a^Model 1 = crude analysis

^b^Model 2 = Model 1 + family income, maternal marital status, maternal skin color and maternal schooling

^c^Model 3 = Model 2 + maternal age, parity and smoking during pregnancy

^d^Model 4 = Model 3 + type of delivery, child’s sex, low birth weight, Apgar at 1st and 5th minute of life, intensive care hospitalization at birth

^e^Model 5 = Model 4 + hospitalization in the 1st year of life, breastfeeding, and maternal depression at the two-year follow-up

Two girls and three boys born at <34 weeks of gestation presented any psychiatric disorder at the age of 6 years (Table 5). At 11 years, three girls and seven boys born at <34 weeks presented any psychiatric disorder. Prevalence of any psychiatric disorder among late preterm girls and boys at 6 years was 12.7 and 15.9%, respectively. At 11 years, late preterm girls and boys had a prevalence of any psychiatric disorders of 12.7 and 17.6%, respectively. There was no difference in prevalence of behavior problems at six and 11 years according to gestational age.

**Table 5:** Prevalence of psychiatric disorders at six and 11 years (any psychiatric disorder, internalizing and externalizing disorders) according to categories of gestational age among singleton pregnancies, Pelotas, Brazil

Gestational age (completed weeks)	6 years							11 years					
	Psychiatric disorders n (%)	*p*-value^a^	Internalizing disordersn (%)	*p*-value^a^	Externalizing disordersn (%)	*p*-value^a^		Psychiatric disordersn (%)	*p*-value^a^	Internalizing disordersn (%)	*p*-value^a^	Externalizing disordersn (%)	*p*-value^a^
Girls													
		0.488		0.706		0.389			0.128		0.436		0.388
<34	2(6.3)		2 (6.3)		0 (0)			3 (9.4)		1 (3.1)		1 (3.1)	
34–36	21 (12.7)		17 (10.3)		5 (3.0)			21 (12.7)		10 (6.1)		8 (4.9)	
37+	142 (10.5)		119 (8.8)		24 (1.8)			109 (8.1)		54 (4.0)		39 (2.9)	
All	165 (10.7)		138 (8.9)		29 (1.9)			133 (8.6)		54 (4.2)		48 (3.1)	
Boys													
		0.324		0.230		0.890			0.143		0.746		0.842
<34	3 (7.0)		1 (2.3)		3 (7.0)			7 (16.3)		1 (2.3)		3 (7.0)	
34–36	28 (15.9)		19 (10.8)		10 (5.7)			31 (17.6)		8 (4.6)		15 (8.5)	
37+	205 (14.3)		135 (9.4)		77 (5.4)			180 (12.5)		69 (4.8)		105 (7.3)	
All	236 (14.3)		155 (9.4)		90 (5.4)			218 (13.2)		78 (4.7)		123 (7.4)	

^a^ *p*-value = Pearson *X* ^2^ test

No association was observed between gestational age and psychiatric disorders neither in crude nor in adjusted analyses (Table 6) at the age of 6 years. At 11 years, the odds ratio for any psychiatric disorder was 60% (1.6; 1.1–2.1) higher among late preterms in comparison to full-term children. At the full adjusted model (Model 5) the highest odds for any psychiatric disorder was seen for late preterm children at 11 years (OR = 1.5; 1.0–2.1), but the confidence interval of the estimate overlapped the null value (Table 6).

**Table 6:** Crude and adjusted odds ratios (OR) with 95% confidence interval (95% CI) for psychiatric disorders at six and 11 years among singleton pregnancies (37+ weeks of gestational age = reference category), Pelotas, Brazil

Gestational age (completed weeks)	Models	6 years			11 years		
		Psychiatric disorders OR (95% CI)	Internalizing disorders OR (95% CI)	Externalizing disorders OR (95% CI)	Psychiatric disorders OR (95% CI)	Internalizing disorders OR (95% CI)	Externalizing disorders OR (95% CI)
	Model 1^a^	*p* = 0.149	*p* = 0.153	p = 0.777	*p* = 0.027	*p* = 0.554	*p* = 0.496
<34		0.4 (0.2; 1.3)	0.4 (0.1; 1.3)	1.1 (0.3; 3.6)	1.3 (0.7; 2.6)	0.6 (0.1; 2.4)	1.0 (0.4; 2.9)
34–36		1.2 (0.9; 1.6)	1.2 (0.8; 1.7)	1.2 (0.7; 2, 1)	1.6 (1.1; 2.1)	1.2 (0.7; 2.0)	1.3 (0.8; 2.1)
37+		1.0 (reference)	1.0 (reference)	1.0 (reference)	1.0 (reference)	1.0 (reference)	1.0 (reference)
	Model 2^b^	*p* = 0.208	*p* = 0.237	*p* = 0.873	*p* = 0.048	*p* = 0.701	*p* = 0.634
<34		0.5 (0.2; 1.2)	0.4 (0.1; 1.3)	1.1 (0.3; 3.3)	1.3 (0.7; 2.6)	0.6 (0.1; 25)	1.0 (0.3; 2.7)
34–36		1.2 (0.8; 1.6)	1.2 (0..8; 1.7)	1.2 (0.7; 2.0)	1.5 (1.1; 2.1)	1.1 (0.7; 1.9)	1.3 (0.8; 2.0)
37+		1.0 (reference)	1.0 (reference)	1.0 (reference)	1.0 (reference)	1.0 (reference)	1.0 (reference)
	Model 3^c^	*p* = 0.209	*p* = 0.235	*p* = 0.905	*p* = 0.043	*p* = 0.663	*p* = 0.601
<34		0.5 (0.2; 1.2)	0.4 (0.1; 1.3)	1.0 (0.3; 3.4)	1.3 (0.7; 2.6)	0.6 (0.1; 2.5)	1.0 (0.3; 2.7)
34–36		1.1 (0.8; 1.6)	1.2 (0.8; 1.7)	1.1 (0.6; 2.0)	1.5 (1.1; 2.1)	1.1 (0.7; 1.9)	1.3 (0.8; 2.0)
37+		1.0 (reference)	1.0 (reference)	1.0 (reference)	1.0 (reference)	1.0 (reference)	1.0 (reference)
	Model 4^d^	*p* = 0.173	*p* = 0.240	*p* = 0.835	*p* = 0.073	*p* = 0.543	*p* = 0.578
<34		0.5 (0.2; 1.3)	0.6 (0.2; 2.0)	1.0 (0.3; 3.7)	1.4 (0.6; 3.2)	1.4 (0.3; 6.9)	0.9 (0.3; 3.1)
34–36		1.2 (0.8; 1.7)	1.3 (0.9; 1.9)	1.2 (0.7; 2.2)	1.5 (1.1; 2.2)	1.4 (0.8; 2.4)	1.3 (0.8; 2.1)
37+		1.0 (reference)	1.0 (reference)	1.0 (reference)	1.0 (reference)	1.0 (reference)	1.0 (reference)
	Model 5^e^	*p* = 0.168	*p* = 0.175	*p* = 0.702	*p* = 0.125	*p* = 0.607	*p* = 0.482
<34		0.6 (0.2; 1.7)	0.8 (0.2; 2.8)	1.1 (0.3; 4.3)	1.4 (0.6; 3.2)	1.6 (0.3; 7.6)	1.0 (0.3; 3.2)
34–36		1.3 (0.9; 1.9)	1.4 (1.0; 2.1)	1.3 (0.7; 2.4)	1.5 (1.0; 2.1)	1.3 (0.7; 2.4)	1.4 (0.8; 2.3)
37+		1.0 (reference)	1.0 (reference)	1.0 (reference)	1.0 (reference)	1.0 (reference)	1.0 (reference)

^a^Model 1 = crude analysis

^b^Model 2: Model 1 + family income, marital status, maternal skin color and maternal schooling

^c^Model 3 = Model 2 + maternal age, parity and smoking during pregnancy

^d^Model 4 = Model 3 + type of delivery, child’s sex, low birth weight, Apgar at 1st and 5th minute of life, intensive care hospitalization at birth

^e^Model 5 = Model 4 + hospitalization in the 1st year of life, breastfeeding, and maternal depression at the fourth-year follow-up (for age 6 years) and at the sixth-year follow-up (for age 11 years)

## Discussion

After controlling for a number of critical confounders, this study found no association between preterm birth and increased risk of behavioral problems at the age of four, 6 and 11 years. The lack of association of gestational age with behavioral problems and psychiatric disorders at three different ages of the same cohort participants reinforces the finding of no association between them.

This finding is in line with results of other studies. [29, 30] In the United States, healthy late-preterm infants were compared with their full-term counterparts from age four through 15 years for numerous standard cognitive, achievement, socio-emotional, and behavioral outcomes. [29] No differences were found between late-preterm and full-term children. Through age 15 years, the mean difference of most of the outcomes hovered around 0.

Also, in a large nationally representative Australian cohort of 5000 children, aged 0 to 1, 2 to 3, and 4 to 5 years of age, children born at 33–36 weeks were compared with healthy-term peers. Child mental health was measured by mother-report on the Strengths and Difficulties Questionnaire. [31] There were small increases in emotional symptoms and total difficulties for the preterm group at age four to 5 years. After adjusting for maternal and socio-demographic factors, preterm birth continued to predict emotional symptoms but not total difficulties at age four to 5 years [30].

Other studies, most of them conducted in high-income countries, have reported higher risks for internalizing and externalizing problems and psychiatric morbidity among the preterm children. [1–3, 32] Contextual factors may play a role at the observed difference between the studies. For instance, an analysis comparing single-born children from the Pelotas 2004 birth cohort at the age of 6 years with children of similar age from the Avon Longitudinal Study of Parents and Children, in the United Kingdom, to investigate whether birth exposures play a causal role in the development of childhood attention problems, showed that whereas Attention Deficit Hyperactivity Disorder (as assessed by means of DAWBA) was associated with preterm birth among children from the British cohort (OR = 2.33, 95% CI 1.23–4.42), there was no association with gestational age in the Pelotas cohort. [33] Compared to gestational age at birth, social, health and economic factors may play a greater role in the development of behavioral problems in middle-income than in high-income settings.

A higher social risk, represented by low parental educational level, younger maternal age, and low socio-economic status, is strongly associated with increased behavior problems. [1, 34–36] Maternal clinical depression and anxiety have a negative influence on mother-infant interaction and this in turn affects the behavioral outcome of the children. [37–39] All these risk factors are more prevalent in low and middle-income settings. Such characteristics of the confounding structure were controlled for in the current study.

Medical factors, such as the length of stay in a neonatal intensive care unit, a prolonged need for artificial ventilation and postnatal corticosteroid exposure may negatively affect behavioral outcome. [1] The access to full intensive care technology and the survival rate after intensive care management are higher among preterm children born in high-income than among those born in low and middle-income settings. Lack of adjustment for such characteristics may positively confound the association between age at birth and behavioral outcomes of the children.

Preterm children that were included in the current analyses are a subsample of all preterm babies that belonged to the cohort. The neonatal mortality rate during the year of 2004 in Pelotas was of 12.7‰ live births [40] (three times higher than that for high-income countries in that year - 4‰). [41] The majority of deaths was due to preterm-related complications. [40] Single babies born at <34 weeks represented 22.3% (125/561) of all preterm births and more than one fifth of them (22%) (28/125) died at the neonatal period whereas 77% (75/97) of the survivors were entered in the current analysis. Moreover, more than half of those born at <28 weeks died in the first week of life (16/25; 64%). The high neonatal and post-neonatal mortality rates among preterm newborns in the present study may have excluded from the analyses the most severely impaired children.

Among the strengths of this study is the large population-based birth cohort with a small rate of losses and refusals in all the waves of follow-up. The instruments employed to measure the outcome (CBCL and DAWBA) were validated for use in the Brazilian population. A number of critical co-variables, including maternal depression and family environmental characteristics, were entered in the adjusted analyses, so minimizing the likelihood of residual confounding that can spuriously lead to associations that are biased due to the lack of control for important confounders.

On the other hand, a limitation is that the study relied only on the mother or the caregiver perspective for the assessment of the children behavioral functioning (the CBCL and DAWBA versions for teachers were not applied). Teachers and parents tend to notice different kinds of behavior problems. [42] At the age of 6 years, many of the cohort children were not yet attending the school and the remaining were at the beginning of the school life. Even so, information on teachers’ complaints regarding the child behavior was gathered from the mother during the six-year and 11-year interview and was used by the Rater when judging for the presence of emotional problem. Additionally, children with different risks may have been inappropriately pooled into the <34 weeks group thus impairing the study capacity of assessing their behavioral outcomes [43].

## Conclusion

There was no association between gestational age and behavioral problems at four, six and 11 years of age among children enrolled in the Pelotas 2004 Birth Cohort and followed-up from birth to 11 years in a middle-income setting. Behavioral problems from middle-childhood to early-adolescence were more related to family socio-economic characteristics than to GA age at birth. This study highlights the importance of targeting children from families with less favorable socio-economic conditions, a risk factor that co-occur with preterm birth in low and middle-income settings, with the aim of preventing behavioral problems in childhood and adolescence.

## Abbreviations

ADHD: Attention Deficit Hyperactivity Disorder
ANOVA: Analysis of variance
CBCL: Child Behavioral Checklist
DAWBA: Development and Well-Being Assessment
ICD-10: International Classification of Diseases-Tenth Revision
LMP: Last menstrual period
SDQ: Strengths and Difficulties Questionnaire
